# Cytokine expression of soft tissue cells cultured with titanium discs and their respective supernatants in vitro

**DOI:** 10.1007/s00784-024-06123-1

**Published:** 2025-01-14

**Authors:** Natália dos Santos Sanches, Layla Panahipour, Lei Wang, Atefe Imani, Caroline Liberato Marchiolli, Lara Cristina Cunha Cervantes, Maria Cristina Ruiz Voms Stein, Sara Alves Berton, Francisley Ávila Souza, Roberta Okamoto, Idelmo Rangel Garcia Júnior, Reinhard Gruber

**Affiliations:** 1 Department of Oral Biology, University Clinic of Dentistry, Medical University of Vienna, 1090 Vienna, Austria; 2 Department of Diagnosis and Surgery, School of Dentistry, São Paulo State University (UNESP), Araçatuba, 16015-050Sao Paulo, Brazil; 3 Wenzhou Institute, University of Chinese Academy of Sciences, Wenzhou, 325000 China; 4 University of Brazil, 15600-000 São Paulo, Fernandópolis Brazil; 5 Department of Basic Sciences, School of Dentistry, São Paulo State University (UNESP), Araçatuba, 16015-050Sao Paulo, Brazil; 6 Austrian Cluster for Tissue Regeneration, 1200 Vienna, Austria; 7 Department of Periodontology, School of Dental Medicine, University of Bern, 3010 Bern, Switzerland

**Keywords:** Odanacatib, Simulated body fluid, Titanium disc, Supernatant, Cell culture techniques, Inflammatory response

## Abstract

**Objective:**

Titanium surface modifications improve osseointegration in dental and orthopedic implants. However, soft tissue cells can also reach the implant surface in immediate loading protocols. While previous research focused on osteogenic cells, the early response of soft tissue cells still needs to be better understood.

**Material and methods:**

We have established a bioassay to this aim where human gingival fibroblasts, HSC2 oral squamous carcinoma cells, and murine bone marrow cells were cultured onto titanium discs or exposed to the respective supernatants for overnight. Modifications were double acid-etching (SLA), and coating with simulated body fluid (SBF) with or without odanacatib (ODN), a selective cathepsin K inhibitor reducing bone resorption.

**Results:**

Our findings indicate that direct contact with titanium discs, with all surface modifications, slightly reduces cell viability. Growing gingival fibroblasts on discs consistently showed a trend toward increased IL8 expression. In HSC2 cells, this setting significantly increased IL1 and IL8 expression, confirmed by the immunoassay. Murine bone marrow macrophages also showed an increase in IL1 and IL6 expressions. Supernatants of the respective discs failed to cause these changes. Although ODN coating inhibited cathepsin K, osteoclastogenesis remained unchanged.

**Conclusions:**

These findings suggest that titanium discs do not provide a favorable in vitro surface for oral soft tissue cells as they lose viability and respond with a moderately increased expression of inflammatory cytokines.

**Clinical relevance:**

The soft tissue surrounding a dental implant can impact rehabilitation success. Understanding how soft tissue cells respond to titanium surface is potentially relevant to understand clinical outcomes.

**Supplementary Information:**

The online version contains supplementary material available at 10.1007/s00784-024-06123-1.

## Introduction

Implant dentistry became a widely established field allowing tooth replacement primarily by titanium screws that are inserted into the alveolar bone after defect healing – thus, the titanium surface is coming into direct contact with the local bone cells [1]. However, immediate implant placement has become a more progressive approach; here, implants are placed immediately after the natural tooth is extracted. Implants are consequently placed in fresh sockets, leaving a micro gap between the titanium surface and the surrounding tissue, which is not restricted to bone cells, but oral soft tissue cells are present [2]. Thus, there is a reason to uncover how not only bone, but also oral soft tissue cells respond to a titanium surface. Osseointegration is even considered a controlled foreign body reaction possibly driving soft tissue mucositis, independent of the classical plaque-induced inflammatory osteolysis, resembling aseptic loosening [3, 4]. Thus, a growing debate exists about how implants' material and surface properties affect this local bone but also soft tissue cells response [5].

Wear particles and ions released from dental implants receive attention as factors modulating peri-implant bone metabolism [6, 7]. Histological specimens and advanced analytical techniques provide insights into what happens at the interface between the implant and the vital implant-supporting tissues [5–7] – but it is mainly the in vitro bioassays that helped us to understand the intimate interaction of the titanium and various coatings, usually in the form of discs, with different cell types including the soft tissue fibroblasts [8–10], epithelial cells [9] and macrophages [11, 12] as well as bone-resorbing osteoclasts [13]. Not surprisingly, and similar to what was traditionally linked in orthopedic research [13, 14], questions arise about the possibility of aseptic inflammation caused by either direct contact or microparticles and ions released into the environment or the peaks, edges, and contaminations that come with surface modifications – and aseptic inflammation may also include the response of soft tissue cells.

Surface modifications include but are not limited to commonly used sandblasting followed by acid etching (SLA) [11]. Coating with simulated body fluid (SBF) to generate a hydroxyapatite layer is feasible [12, 15]. Surface coating can also include drug applications, as we have recently shown for odanacatib (ODN; manuscript in review), a selective inhibitor of cathepsin K, which demonstrates efficacy in reducing bone resorption and favors osseointegration [16–18]. All these modifications can be tested by growing the various cell types together with the titanium discs; preparing supernatants from the discs to be used in a bioassay has not been considered so far. There is, thus, the dilemma that we lack bioassays comparing the response of cells, including those of the oral soft tissue, that are either cultured onto titanium discs or with the respective supernatants – allowing us to distinguish a direct from an indirect cell response to the titanium discs – which is the overall aim of the present research.

Titanium disc research provided valuable insights into how, for instance, gingival fibroblasts respond to the new surface. However, information on the expression changes of inflammatory cytokines is scarce. With human gingival fibroblasts, SLA moderately decreased IL6 and increased IL8 expression compared to a regular tissue culture surface [8]. Even systematic reviews relate the macrophage inflammatory response to potential implant surfaces in vitro [19]. For instance, when compared to tissue culture-treated coverslips, an SLA surface increased IL1 expression in RAW 264.7 murine macrophage cells [20] The same is true concerning TNFα release by RAW 264.7 cells in culture with SLA surface-treated titanium discs [21]. Again, there is a moderate increase of IL1 and TNFα in primary murine macrophages [22]. Since osteoclasts originate from the monocytic lineage, titanium discs with SLA treatment were assessed for their potential to modulate the process of osteoclastogenesis [23]. Moreover, even though SBF is widely used to coat titanium surfaces [15, 24], there is no research on how the surface coating affects the expression changes of inflammatory cytokines, and the presence of ODN is innovative and new. We can conclude from the existing knowledge that most of the research on how titanium discs and their modifications affect inflammatory gene expression is based on macrophages, which is not surprising as they are professional inflammatory cells, at least the M1 phenotype. However, we have learned from recent single-cell RNAseq research that in periodontitis [25] and periimplantitis [26], it is the fibroblasts and the keratinocytes/epithelial cells [27] – what we consider oral soft tissue cells—that are significant drives of the inflammatory tissue response. It is thus essential to propose fibroblasts as well as epithelial cell lineage cells for titanium disc research in the contact of cytokine expression, considering the potential differential response of the cell being cultured with titanium discs and the respective supernatant.

Supernatants of titanium discs are usually not used for testing the response of the cells to dental implants; however, we consider this setting as a control for the release and potential implication of loosely attached titanium particles and ions, including those of the SBF-coated surface. Moreover, since ODN is a drug that is released from the SBF, it is necessary to rule out the unlikely event of an inflammatory response. Pure titanium particles increase the expression of cytokines, including IL8 and potentially also IL6 in oral fibroblasts [28, 29] and IL6 and PGE2 in fibroblasts from the periprosthetic membrane [30]; thus, the supernatant of titanium particles may cause cytokine expression changes. Considering SBF, coating of titanium discs with amorphous or crystalline hydroxyapatite (HA) had no significant impact on TNFα expression by human peripheral blood mononucleated cells [12]. However, HA particles raise IL6 in the foreskin and dermal fibroblasts, but neither IL1 nor TNFα were identified in the culture medium at any particle concentration investigated [31, 32]. Thus, understanding the impact of hydroxyapatite coating on the cell response to titanium discs and the respective supernatants required further knowledge. Also, ODN is potentially relevant in changing the expression of cytokines. Initially developed to treat postmenopausal osteoporosis based on inhibition of osteoclastic cathepsin K [16, 17], ODN prevents inflammation and bone loss from endodontic disease [33]. Its ability to decrease IL6 and TNFα through the modulation of macrophage activity highlights its expanded therapeutic potential [33]. Despite its clinical improvement being halted due to an increased risk of stroke observed in the Long-Term Odanacatib Fracture Trial (LOFT; ClinicalTrials.gov NCT00529373; Protocol MK-0822–018) Phase III clinical trial [18], local application methods, such as coating dental implants with ODN, are improving osseointegration in a rat implantation model (unpublished). There is a demand for in vitro bioassays, including the supernatants of the original titanium discs.

The present research aims to compare the cytokine expression of various soft tissue cell types—including the HSC2 epithelial cell line, apart from fibroblasts and bone marrow cells – grown in the presence of the titanium discs with their surface modifications SBF and ODN, and the respective supernatants. The selection of cells was based on the involvement in early implant integration [34, 35]; HSC2 cells represent oral epithelial cells, with the limitation that they are of tumor origin and perhaps do not fully reflect the behavior of primary oral epithelial cells [36], gingival fibroblasts indicating a response of mesenchymal cells in general, and macrophages being central to innate immunity. This approach allows us to distinguish between direct cellular responses to the discs and indirect cellular responses elicited by exposure to the corresponding supernatants. Our data suggests that our established HSC2 oral squamous carcinoma cell line [37] increasingly expresses IL1 and IL8 when grown with discs but not with the supernatants. In murine bone marrow cultures, the discs, but not the respective supernatants, caused a moderate IL1 expression increase in murine macrophages [20]. However, they failed to consistently change osteoclastogenesis, as indicated by the formation of multinucleated expressing TRAP and cathepsin K. The present research is thus a primer for future studies that consider not only direct contact but also the use of supernatants in biomaterial testing.

## Materials and methods

### Coating of the titanium discs and topographic characterization

The manufacturer carried out SLA treatment of titanium disc (11 mm diameter, 3.0 mm length; Implalife®, Medical-Dental Products Industry, Jales, São Paulo, Brazil) following a patented protocol. Subsequently, discs underwent surface coating with SBF, with and without ODN at a concentration of 260 µg/ml (SBF + ODN). The titanium discs were immersed in 50 mL of a 5.0 mol/L NaOH (Sigma-Aldrich, St. Louis, MO, USA) solution and incubated at 60 °C for 24 h to chemically activate their surfaces. Subsequently, the simulated body fluid (SBF) was prepared as described by Aparecida [38]. This solution replicates the ionic composition and pH of human blood plasma, providing a biomimetic environment. The discs were immersed and incubated in SBF containing either no or 260 µg/mL of odanacatib (ODN, MedChemExpress LLC, Monmouth Junction, NJ) at pH 7.25 and 37 °C for 4 days. To maintain the ionic balance and ensure stability, the solutions were replaced every 24 h throughout the incubation period. Subsequently, the titanium discs were washed in a 10% aqueous hydrogen peroxide solution (Sigma-Aldrich) for 4 h, with solution changes every hour at room temperature. This was followed by a quick wash in 0.9% sodium chloride solution and absolute alcohol. After the final dry in each titanium disc was individually packaged and sterilized using gamma radiation at a dose of 25 kGy, performed by Implalife®. This sterilization dose ensures the effective elimination of microorganisms while preserving the structural and chemical integrity of the titanium. Morphological and surface properties were analyzed using scanning electron microscopy with an EVO LS15 instrument (Zeiss, Jena, Germany), coupled with an energy-dispersive X-ray spectroscopy (EDX) system for the semi-quantitative analysis of chemical composition and elemental mapping on the surfaces. Contact angle measurements were performed at room temperature and 75% relative humidity with an OCA 15Plus optical instrument (Dataphysics Instruments GmbH, Filderstadt, Germany), using a dispensing volume of 6.5 μl at a rate of 5.00. Three measurements per sample were taken and average values were calculated.

### Surface roughness assessment

Surface roughness was measured using a portable roughness meter (SJ-410, Mitutoyo Sul Americana Ltda, Santo Amaro, SP, Brazil). Measurements were made in three specific regions of the disc surface: right, center and left. For each region, the roughness parameters (Ra) and (Rz) were recorded, with Ra being the arithmetic mean of the absolute deviations of the mean line of the roughness profile and Rz the mean of the maximum heights. The measurements followed a straight line, using a cut-off of 0.25 mm, with constant speed of 0.05 mm/s for 12 s. The values originally obtained in Angström (Å) were converted to the nanometric scale (nm), and the average of the four measurements performed per sample was tabulated for analysis.

### Preparation of supernatants from titanium discs

Supernatants for gingival fibroblast, oral squamous cell carcinoma cell line (HSC2), and macrophage stimulation were prepared by incubating one disc from each group in 2 ml of serum-free Dulbecco's Modified Eagle Medium (DMEM; Sigma-Aldrich, St. Louis, MO, USA) with 1% antibiotics (PS; Sigma-Aldrich, St. Louis, MO, USA). For osteoclastogenesis, supernatants were prepared with Minimum Essential Medium Eagle-Alpha Modification (αMEM; Invitrogen, Grand Island, NY, USA) with 1% antibiotics and 10% fetal calf serum (FCS; Bio&Sell). One disc was placed per 15 ml tube on an orbital shaker at 450 rpm and room temperature. The supernatants were collected after 12 h and directly transfected to 1.5 mL tubes. Supernatants were stored at 4 °C for not longer than 48 h prior to the cell exposure.

### Gingival fibroblasts and oral squamous carcinoma cells

Human gingival fibroblasts were isolated from gingival explants derived from extracted wisdom teeth, following the patients' informed consent. The local Ethical Committee approved the protocol (EK Nr. 631/2007). The oral squamous cell carcinoma cell line (HSC2) was obtained from the Health Science Research Resources Bank (Sennan, Japan). All cells were cultured in a humidified atmosphere at 37 °C with 5% CO_2_ in a growth medium consisting of DMEM, supplemented with 1% antibiotics and 10% fetal calf serum (FCS; Capricorn Scientific GmbH, Ebsdorfergrund, Germany). Gingival fibroblasts were seeded at a density of 1 × 10^5^ cells/cm^2^, and HSC2 cells were seeded at 2 × 10^5^ cells/cm^2^ into 24-well plates (VWR International, Radnor, PA, USA) containing a disc for each group respectively. The following day, cells were exposed to 10 ng/mL IL1β and TNFα (ProSpec Tany Techno Gene Ltd., Ness-Ziona, Israel) as a positive control for inflammation induction, as well as to the undiluted supernatant of the disc for each group respectively. After an overnight incubation under the same culture conditions, gene expression analysis was performed from the RNA, and the supernatant was collected for immunoassay.

### Murine bone marrow-derived macrophages and osteoclasts

Bone marrow-derived macrophages and osteoclasts were obtained from 6–8-week-old BALB/c mice acquired from Animal Research Laboratories, Himberg, Austria. Bone marrow cells were harvested from the femora and tibiae and seeded at a 4 × 10^6^ cells/cm^2^ density into 24-well plates. The cells were cultured for 7 days in αMEM with 1% antibiotics, supplemented with 10% FCS, and 20 ng/mL macrophage colony-stimulating factor (M-CSF; Prospec, Ness-Ziona, Israel) to induce macrophage differentiation. On day 7, the cells were exposed to 5% saliva as a positive control for inflammation induction and to the undiluted supernatant of the disc and the disc for each group, respectively. After an overnight incubation under the same culture, gene expression analysis was performed on the RNA, and the supernatants were collected for immunoassay. To promote osteoclast differentiation, bone marrow cells were seeded at a density of 4 × 10^6^ cells/cm^2^ into both 24-well and each containing a disc for the respective groups, and 48-well plates (CytoOne, Starlab International, Hamburg). They were cultured for 5 days in αMEM supplemented with 10% FCS and 1% antibiotics under the same culture conditions. Osteoclastogenesis was induced by the addition of receptor activator of nuclear factor kappa-B ligand (RANKL, 30 ng/mL), M-CSF (20 ng/mL), and human TGF-β1 (10 ng/mL; Prospec, Ness-Ziona, Israel). Additionally, the undiluted supernatant of the disc was added for each group. Histochemical staining for TRAP was carried out according to the manufacturer's instructions (Sigma-Aldrich). Furthermore, the gene expression of TRAP and CTSK was analyzed.

### Viability assay

Viability experiments were performed for gingival fibroblasts and HSC2 which were incubated overnight in 24-well plates. Following this incubation, the cells were stimulated with either the undiluted supernatants or titanium discs from each group overnight. MTT (3-[4,5-dimethylthiazol-2-yl]−2,5-diphenyltetrazolium bromide; Sigma) was then added to each well at a final concentration of 0.5 mg/mL and incubated for 2 h at 37 °C. The medium was removed, the formazan crystals were solubilized with dimethyl sulfoxide (Sigma-Aldrich), and the solution was transferred to a 96-well plate (CytoOne, Starlab International, Hamburg). Optical density was measured at 570 nm, and the data were normalized to unstimulated control values. Cell viability was further confirmed using a live-dead colorimetric assay kit, following the manufacturer’s instructions (Enzo Life Sciences, Inc., Lausanne, Switzerland).

### Scratch assay

The scratch assay was performed on gingival fibroblasts and HSC2 cells, which were seeded at densities of 1 × 10^5^ cells/cm^2^ and 2 × 10^5^ cells/cm^2^, respectively, into 6-well plates. After overnight incubation, a sterile plastic micropipette tip was used to create a uniform scratch in each well. The cells were then washed with serum-free medium to remove debris and exposed either the undiluted supernatant or titanium discs from each group from 0 to 12 h.

### Reverse transcription quantitative real-time PCR (RT-qPCR) and immunoassay

For RT-qPCR, total RNA was isolated after stimulation with the ExtractMe total RNA kit (Blirt S.A., Gdansk, Poland) followed by reverse transcription and polymerase chain reaction (LabQ, Labconsulting, Vienna, Austria) on a CFX Connect Real-Time PCR Detection System (Bio-Rad Laboratories, Hercules, CA, USA). The mRNA levels were determined by normalizing to the housekeeping gene GAPDH using the ΔΔCt method. The primer sequences for the target genes are indicated in Table 1 and Table 2. For the immunoassay, Quantikine ELISA kit for mouse IL1 and human CXCL8/IL8 were used (R&D Systems, Minneapolis, MN).

**Table 1:** Human primer sequence

Gene	Forward (5′ 3’)	Reverse (3′ 5’)
hIL1β	ATGATGGCTTATTACAGTGGCAA	GTCGGAGATTCGTAGCTGGA
hIL8	AACTTCTCCACAACCCTCTG	TTGGCAGCCTTCCTGATTTC
hGAPDH	AAGCCACATCGCTCAGACAC	GCCCAATACGACCAAATCC

**Table 2:** Mouse primer sequence

Gene	Forward (5′ 3’)	Reverse (3′ 5’)
mIL1α	TTGGTTAAATGACCTGCAACA	GAGCGCTCACGAACAGTTG
mIL6	GCTACCAAACTGGATATAATCAGGA	CCAGGTAGCTATGGTACTCCAGAA
mTRAP	CGTCTCTGCACAGATTGCAT	AAGCGCAAACGGTAGTAAGG
mCTSK	TGTATAACGCCACGGCAAA	GGTTCACATTATCACGGTCACA
mGAPDH	AACTTTGGCATTGTGGAAGG	GGATGCAGGGATGATGTTCT

### Immunofluorescence analysis

Gingival fibroblasts and HSC2 cells were seeded onto Millicell EZ slides (Merck KGaA, Darmstadt, Germany) at densities of 0.5 × 10^5^ cells/cm^2^ and 1 × 10^5^ cells/cm^2^, respectively. The cells were treated with the undiluted supernatants from discs of each group for one hour. Following treatment, the cells were fixed with 4% paraformaldehyde, blocked using 1% bovine serum albumin (Sigma-Aldrich, St. Louis, MO, USA), and permeabilized with 0.3% Triton X-100 (Sigma-Aldrich, St. Louis, MO, USA). The primary antibody NF-κβ P65 (Cell Signaling Technology, Cambridge, UK) was added and incubated overnight at 4 °C. Detection was carried out using Alexa 488 secondary antibody (CS-4412, Cell Signaling Technology). Fluorescent images were obtained using a microscope with a DAPI-FITC dual excitation filter (Echo Revolve Fluorescence Microscope, San Diego, CA, USA).

### Western blot

Gingival fibroblasts and HSC2 cells were seeded at densities of 2 × 10^5^ cells/cm^2^ and 2.5 × 10^5^ cells/cm^2^ in a 6-well plate, respectively. After serum starvation overnight, the cells were exposed to the undiluted supernatants from SLA discs, and IL1β + TNFα as positive control for one hour. Cell lysates were prepared using SDS buffer with protease and phosphatase inhibitors (complete ULTRA Tablets and PhosSTOP; Roche, Mannheim, Germany), separated by SDS–PAGE, and transferred onto PVDF membranes (Roche Diagnostics, Mannheim, Germany). The membranes were blocked and incubated with primary antibodies targeting phospho-p38 (E1) and total p38 (C-20) (sc-166182 and sc-535; Santa Cruz Biotechnology, Inc., Dallas, TX). For detection, HRP-conjugated secondary antibodies (CS-7074; Cell Signaling Technology, Cambridge, UK) were applied. After a 5-min incubation with Clarity Western ECL Substrate (Bio-Rad Laboratories, Inc., Hercules, CA, USA), chemiluminescence signals were captured using a Chemi Doc imaging system (Bio-Rad Laboratories). Finally, densitometric analysis of the blots was performed with Image Lab software (Bio-Rad Laboratories).

### Cathepsin K activity bioassay on titanium discs

Lysis of osteoclastic bone marrow cultures was conducted using 0.1% Triton X-100 (Sigma-Aldrich) in PBS in a 6-well plate. TRAP staining confirmed osteoclast formation. Discs were coated with osteoclastic lysate and incubated for 2 h under agitation. The procedure was following the manufacturer’s protocol (BML-AK430; Enzo Life Sciences), incorporating 0.01 mM CatK substrate II (Sigma-Aldrich). The bioassay was performed in a white 96-well microplate. Fluorescence intensity (λ_exc = 360 nm; λ_em = 460 nm) was measured every 15 min for 2 h using a microplate reader with automatic gain adjustment.

### Statistical analysis

**Fig. 1:**
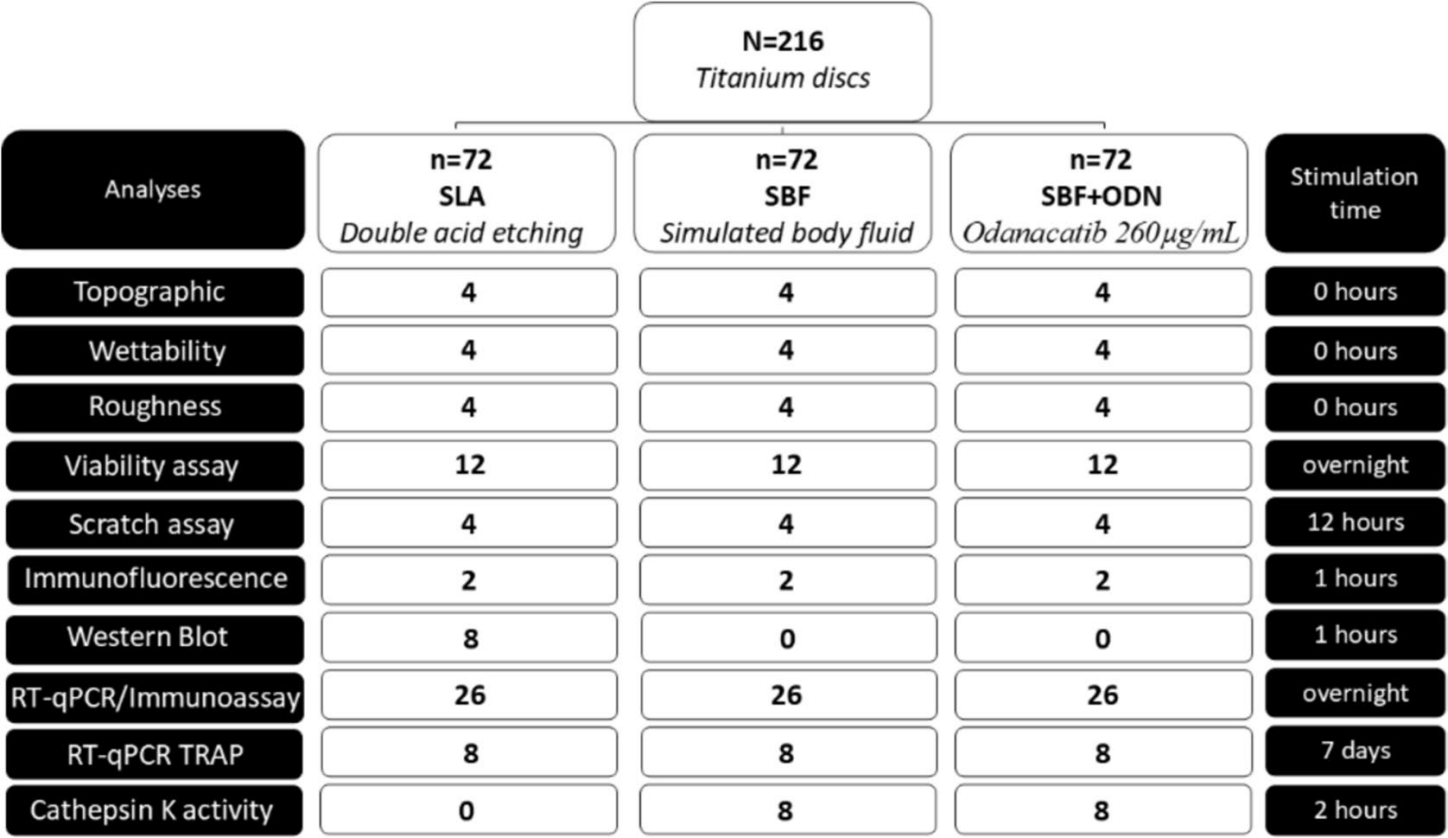
Workflow represents the sample distribution of 216 titanium discs in three groups of surface treatment: SLA, SBF and SBF + ODN. The SLA was considered as a control group. The diagram describes the analyses performed and the respective exposure times for each experiment

All experiments were conducted at least three times. Each data point represents an independent experiment, with samples obtained individually from discs and their respective supernatants for each group. The workflow is shown in Fig. 1 representing the sample used for the experiment. Statistical analysis was performed using a ratio-paired t-test for single comparisons. All analyses were performed out using Prism V9 (GraphPad Software, La Jolla, CA, USA), with significance set at p < 0.05.

## Results

### Surface topography and chemical properties of titanium discs

**Fig. 2:**
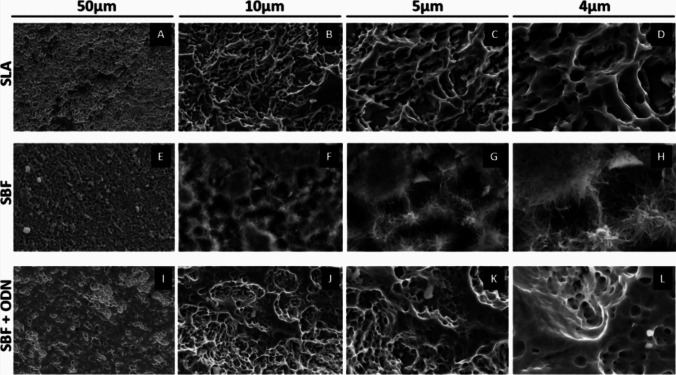
Scanning electron microscopy (SEM) images of the surfaces of SLA, SBF, and SBF + ODN discs at 10,000 × magnification. **A**-**D** SLA shows uniform microroughness with varying depths and sizes. **E**–**H** SBF exhibits the expected spider web-like pores of the hydroxyapatite coating. **I**-**L** SBF + ODN displays a rough texture with globular microcavities, interlocking pores, and nanometric particles. Obviously, the presence of ODN affects the morphology of the hydroxyapatite coating compared to the SBF alone discs – and its wettability

**Fig. 3:**
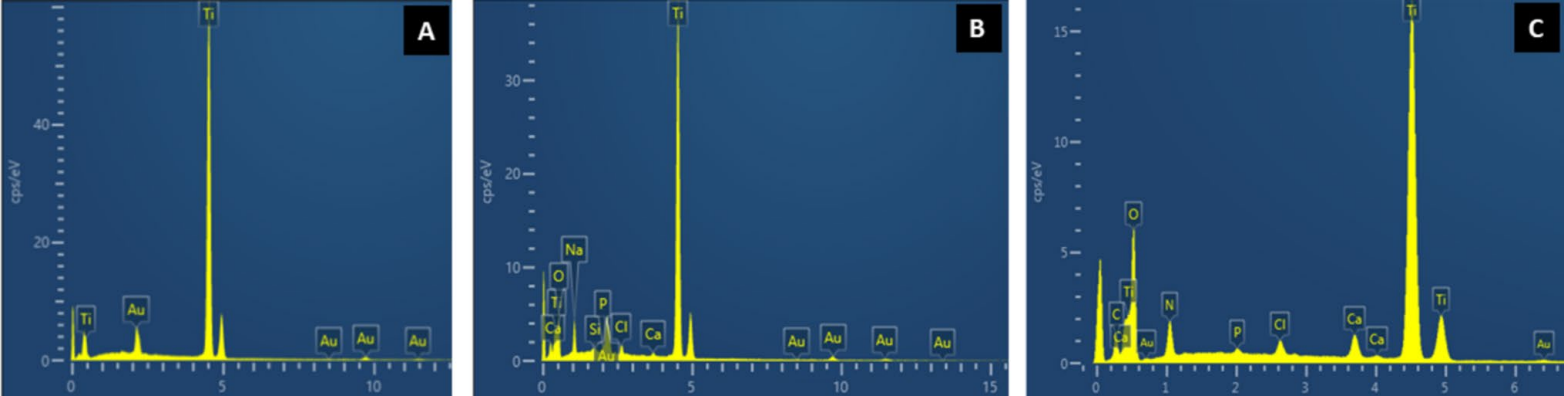
Energy-dispersive X-ray (EDX) spectroscopy of the surfaces of SLA, SBF, and SBF + ODN discs with a gold (Au) as a coating for analysis. As expected, **A** the SLA surface shows prominent titanium (Ti) peaks, and (**B**) the SBF surface displays peaks corresponding to calcium (Ca) and phosphorus (P). **C** The SBF + ODN surface exhibits peaks for carbon (C), nitrogen (N), and oxygen (O), corresponding to the ODN components, in addition to the calcium and phosphorus peaks

**Fig. 4:**
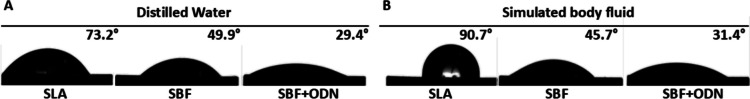
Image captured by the video-based optical contact angle measuring instrument for each group—SLA, SBF, and SBF + ODN with test solutions: **A** distilled water and **B** simulated body fluid solution. The SLA surface was the least hydrophilic for the distilled water and the simulated body fluid solution. The most hydrophilic surface was SBF + ODN, significantly more than SBF alone

**Fig. 5:**
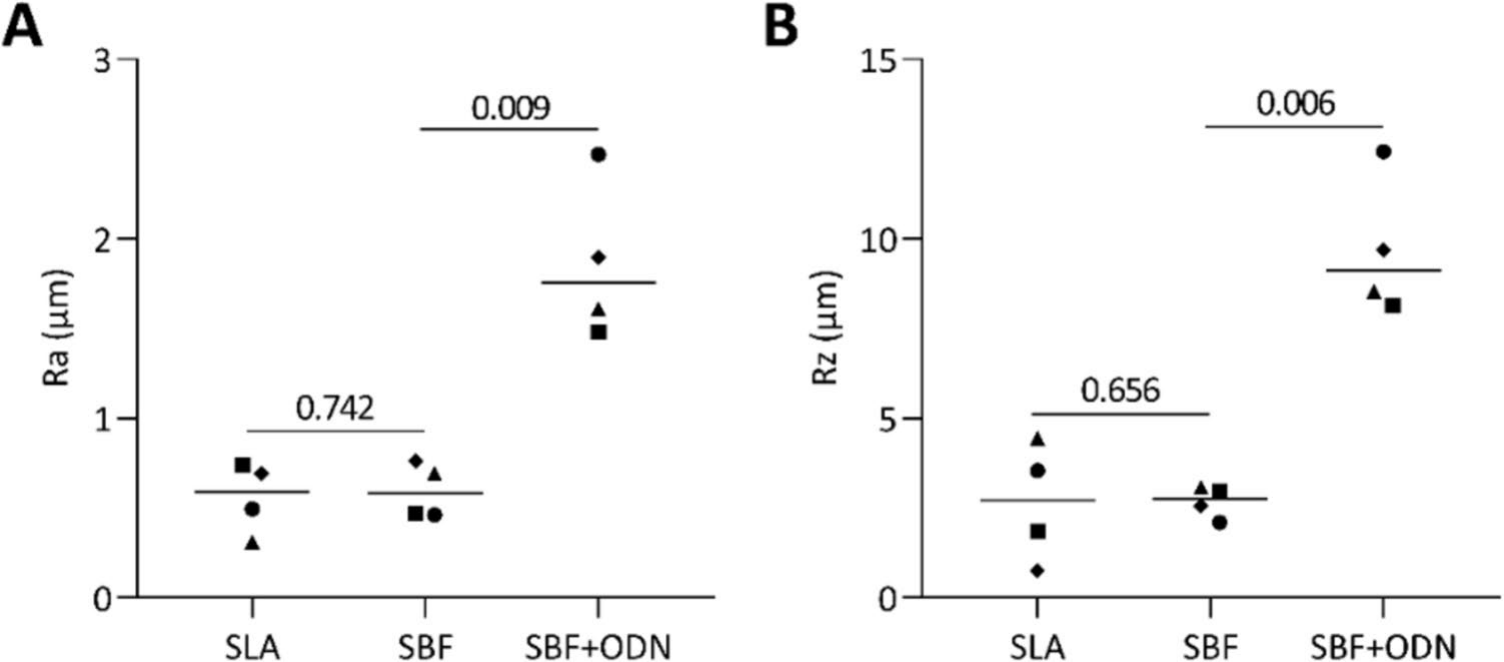
Surface roughness was evaluated by measuring Ra (**A**) and Rz (**B**) values for each group SLA, SBF and SBF + ODN. The results showed that the ODN coating significantly increased surface roughness compared to SBF alone. Different symbol shapes represent mean values of independent readings. Statistical analysis was performed using ratio-paired t-tests compared to untreated controls, and p-values are shown

The topographic properties and the wettability of a titanium surface impact cell interaction during the early stages of osseointegration [39]. To assess topography, SEM analysis revealed that the SLA surface, as we expected, exhibited a subtractive topography pattern, resulting in microroughness with varying depths and sizes (Fig. 2A-D). The SBF coating displayed a uniform roughness with a spider web-like hydroxyapatite surface (Fig. 2E-H). Interestingly, the presence of ODN had a significant impact on SBF coating, producing a moderately rough surface with no hallmarks of a hydroxyapatite surface (Fig. 2I-L). The EDX analysis did not indicate any contamination on the surface of the titanium discs. As expected, the SLA treatment showed clear titanium (Ti) peaks (Fig. 3A, Table 3). The SBF alone revealed peaks corresponding to calcium (Ca) and phosphorus (P), indicating the chemical deposition of calcium phosphate (CaP) (Fig. 3B, Table 3) [40]. In the SBF + ODN treatment, carbon (C), nitrogen (N), and oxygen (O) were observed, corresponding to the components of the ODN, alongside the presence of Ca and P, but these were less prominent (Fig. 3B, Table 3). The evaluation of the contact angle follows a similar path where the wettability of the SLA surface was significantly reduced by the SBF coating and even more in the presence of ODN (Fig. 4, Table 4). Furthermore, regarding surface roughness, we observed that the SLA and SBF surfaces exhibited similar roughness values (Figs. 5A and B). However, the SBF + ODN coating resulted in a significantly rougher surface compared to the SBF surface alone (Figs. 5A and B). Therefore, what became apparent is that the presence of ODN in the SBF changes the topographic properties, wettability and roughness.

**Table 3:** Energy-dispersive X-ray (EDX) spectroscopy of the surfaces of SLA, SBF, and SBF + ODN discs, showing the apparent concentration of chemical elements present on the surface

Titanium disc surface	Ti	Ca	P	C	O	N	Cl
SLA	103.65	0	0	0	0	0	0
SBF SBF + ODN	41.04 64.99	15.21 6.27	11.10 0.85	0 6.27	11.20 11.64	0 3.99	1.23 0.45

**Table 4:** Variation in contact angle values (θ) in different surface modifications

Groups	SLA	SBF	SBF + ODN	*P* value
Distilled water	73.20 ± 17.50	49.90 ± 5.00	29.40 ± 0.90	*P* = 0.073 *P* = 0.001
Simulated body fluid	90.30 ± 19.20	45.70 ± 11.80	31.40 ± 9.30	*P* = 0.005 *P* = 0.065

The wetting properties of the discs were evaluated in quadruple under ambient temperature conditions. The data represents the mean ± SD relative to the control. Statistical analysis was performed using ratio-paired t-tests, and the p-values are shown.

### Titanium disc, not supernatant, impairs cell viability in gingival fibroblasts and oral squamous cell carcinoma

**Fig. 6:**
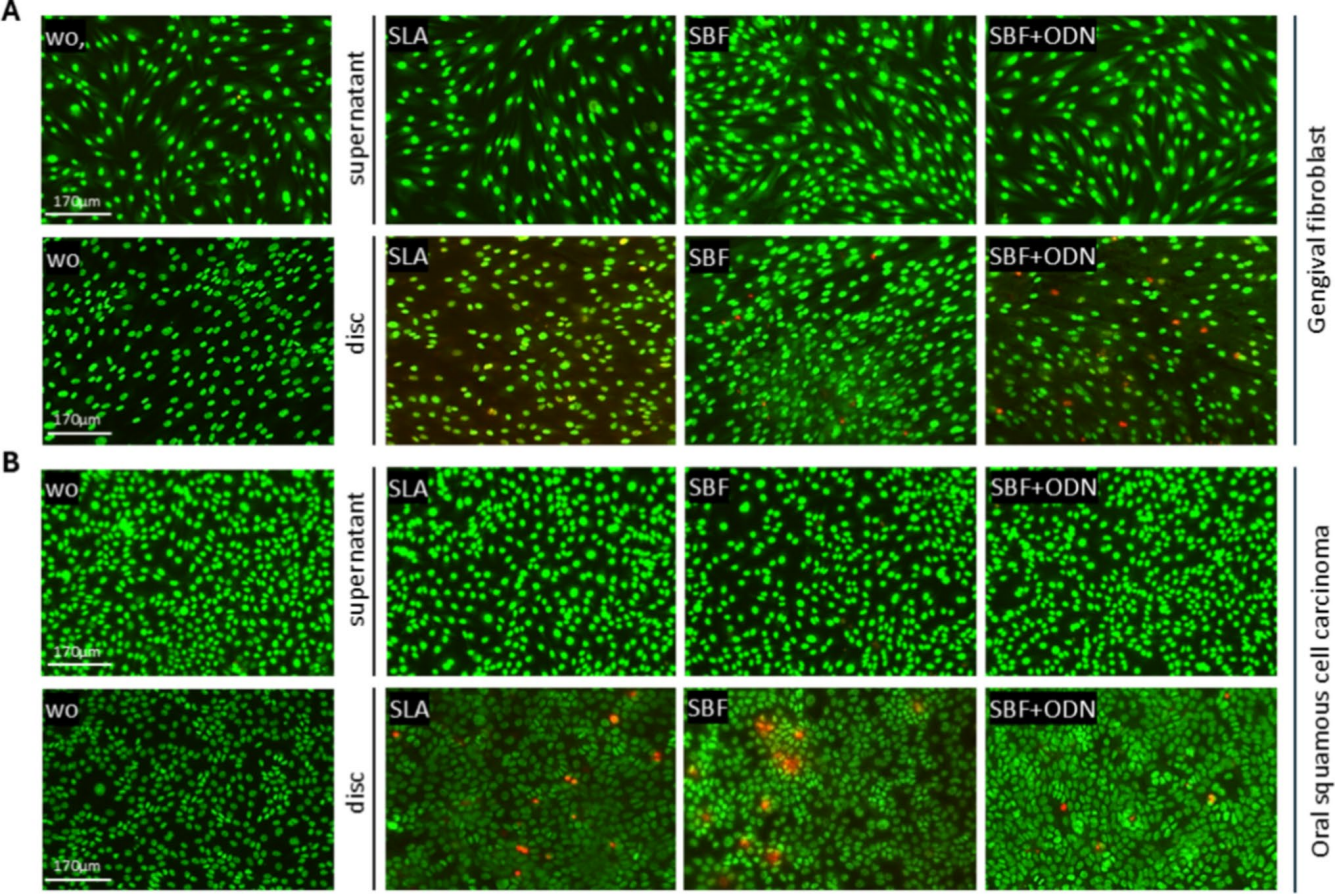
Life–dead staining of gingival fibroblasts (**A**) and HSC2 cells (**B**) exposed overnight to the supernatants or grown on the titanium discs. Viable cells are stained green, while dead cells are stained red. The images suggest that direct contact with the disc surfaces resulted in the irregular appearance of dead gingival fibroblasts and HSC2 cells. In contrast, the supernatant predominantly maintained cell viability. “wo” stands for “without” and shows the untreated cells. Scale bar: 170 μm

Even though titanium implants have widespread clinical use, concerns about cellular adverse responses to the surface have been raised [41]. Therefore, we aimed to investigate the response of cells grown in the presence of supernatants of titanium discs or directly seeded onto the discs. First, we evaluated the impact of the titanium discs with all the surface modifications on the formazan formation, a test for cell number and metabolic activity. Notably, the formazan formation only slightly decreased when gingival fibroblasts and HSC2 cells were exposed to the supernatants of the titanium discs, with no preference for surface treatment. In contrast, however, growing cells in the presence of the titanium discs, and, in particular, when coated with SBF, but independent of ODN, considerably lowered formazan formation. Thus, these results suggested that the effects of the titanium discs on cells are mainly direct, as supernatants cause neglectable changes (Table 5; Supplementary Fig. 1). To better understand how the titanium discs impact cell viability, we used an established life-dead staining assay, which enables the visualization of viable and dead cells appearing in green and red, respectively. Consistent with the formazan formation assay, life-dead staining revealed a noticeable fraction of cells staining red when grown on the surface of the titanium discs, while no obvious red gingival fibroblasts and HSC2 cells could be identified in the presence of the supernatants of the titanium discs, not when coated with SBF and ODN (Fig. 6A and B). The formazan formation, together with the life/dead staining, further underscored a direct impact of a titanium surface on cell viability. Collectively, these findings point towards a possible adverse effect that is associated with an alarming response indicated by the expression of inflammatory cytokines. Additionally, in a scratch test, the results support the HSC2 that the supernatant and the SBF + ODN-coated disc enhanced wound closure (Supplementary Fig. 2), while gingival fibroblasts remained unchanged.

**Table 5:** Cell viability assays were performed on human gingival fibroblasts and HSC2 cells stimulated with the supernatants and discs from each group—SLA, SBF, and SBF + ODN overnight

Cell type	SLA supernatant	SBF supernatant	SBF + ODN supernatant	SLA disc	SBF disc	SBF + ODN disc
Gingival fibroblasts	93.2 ± 3.6	89.2 ± 2.9	90.0 ± 3.2	98.2 ± 4.1	68.2 ± 0.7	97.0 ± 9.5
HSC2	97.4 ± 5.3	84.9 ± 1.6	89.4 ± 1.6	91.4 ± 4.2	49.1 ± 2.2	50.2 ± 5.2

Viability was assessed by measuring formazan production and expressed as a percentage relative to unstimulated controls which are considered as 100% viability. Results showed a significant reduction in cell viability in gingival fibroblasts and HSC2, upon direct contact with the titanium discs. In contrast, the exposure to the supernatants led to only a slight decrease.

### Titanium coating increases IL8 in gingival fibroblasts and oral squamous cell carcinoma

**Fig. 7:**
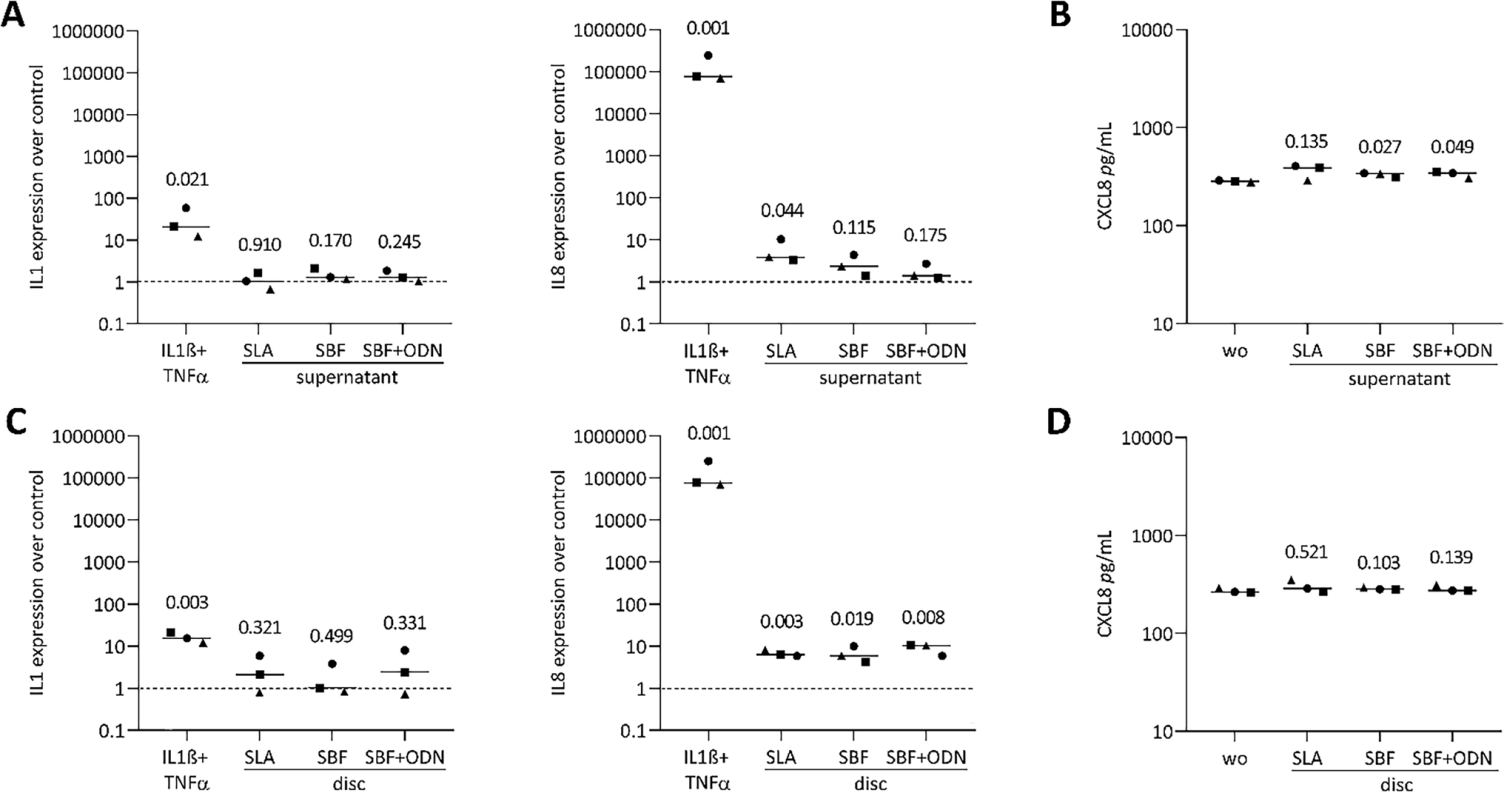
Gingival fibroblasts were exposed to the supernatants (**A**) or grown in the presence of titanium discs (**C**) of each group and IL1β + TNFα as positive control. Results were normalized for expression changes for untreated cells. (**B**, **D**) Immunoassay indicated basal IL8 levels in pg/mL of untreated cells. Different symbol shapes represent independent experiments. Statistical analysis was performed using ratio-paired t-tests compared to untreated controls, and p-values are shown

**Fig. 8:**
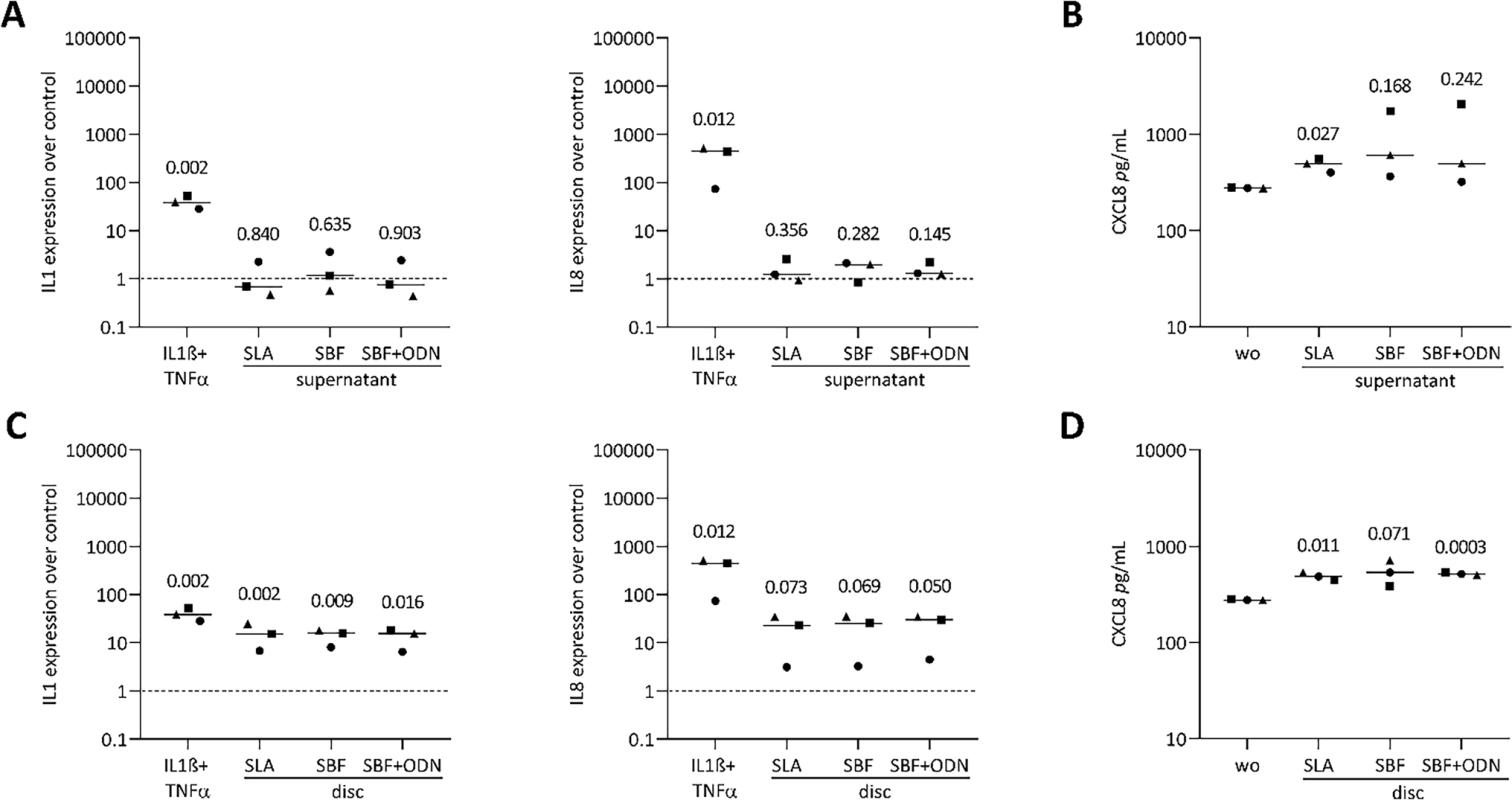
HSC2 cells were exposed to the supernatants (**A**) or grown in the presence of titanium discs (**C**) of each group and IL1β + TNFα as positive control. Results were normalized for expression changes for untreated cells. (**B**, **D**) Immunoassay indicated basal IL8 levels in pg/mL of untreated cells. Different symbol shapes represent independent experiments. Statistical analysis was performed using ratio-paired t-tests compared to untreated controls, and p-values are shown

Given the potential role of dying oral cells in triggering an inflammatory response [9, 42] we compared the expression of lead cytokines in gingival fibroblasts and HSC2 cells under the two experimental conditions – the exposure to undiluted supernatants and the cultures with titanium discs. The gene expression analysis revealed a significant increase of IL8 expression when gingival fibroblasts were cultures with titanium discs (Fig. 7C, but only marginal changes, if at all, when grown the presence of the supernatants (Fig. 7A). We could further validate the expression of IL8 on the protein level by immunoassay (Fig. 7B - D). Although the SBF and SBF + ODN coated discs similarly elevated IL8 expression, this effect did not translate to the protein level (Fig. 7A - C). Similar results were observed in HSC2 cells, where all three coatings led to an increase in IL8 expression, both on the discs and in their respective supernatants, as confirmed by high levels of IL8 protein (Fig. 8B - D). Additionally, IL1 expressions were upregulated across the three surface treatments in HSC2 cells. We also noticed a trend toward increased expression of CXCL1, CXCL2, CXCL10, and IL6 in both cell types when exposed to supernatants and the titanium discs. However, these changes were not consistently observed across all experiments (Supplementary Fig. 3 and 4). Overall, the effects of the surface coatings and supernatants on IL1 and IL8 expression were less pronounced compared to the more vigorous activity of IL1β and TNFα. Consistent with the established role of NFκβ signaling pathways in IL8 expression [42, 43], exposure to undiluted supernatants did not affect the nuclear translocation of NFκβ-p65 in either gingival fibroblasts or HSC2 cells (Supplementary Fig. 5). Taking together these results, we further investigated additional signaling pathways that may contribute to IL8 expression, with a specific focus on p38 MAPK signaling. Notably, the supernatant from SLA discs enhanced p38 phosphorylation in gingival fibroblasts (Supplementary Fig. 6). These findings indicate that the p38 signaling pathway plays a partial role in mediating IL8 expression induced by SLA supernatant in gingival fibroblasts, while this effect was not observed in HSC2 cells.

### Titanium coating increases IL1 in macrophage

**Fig. 9:**
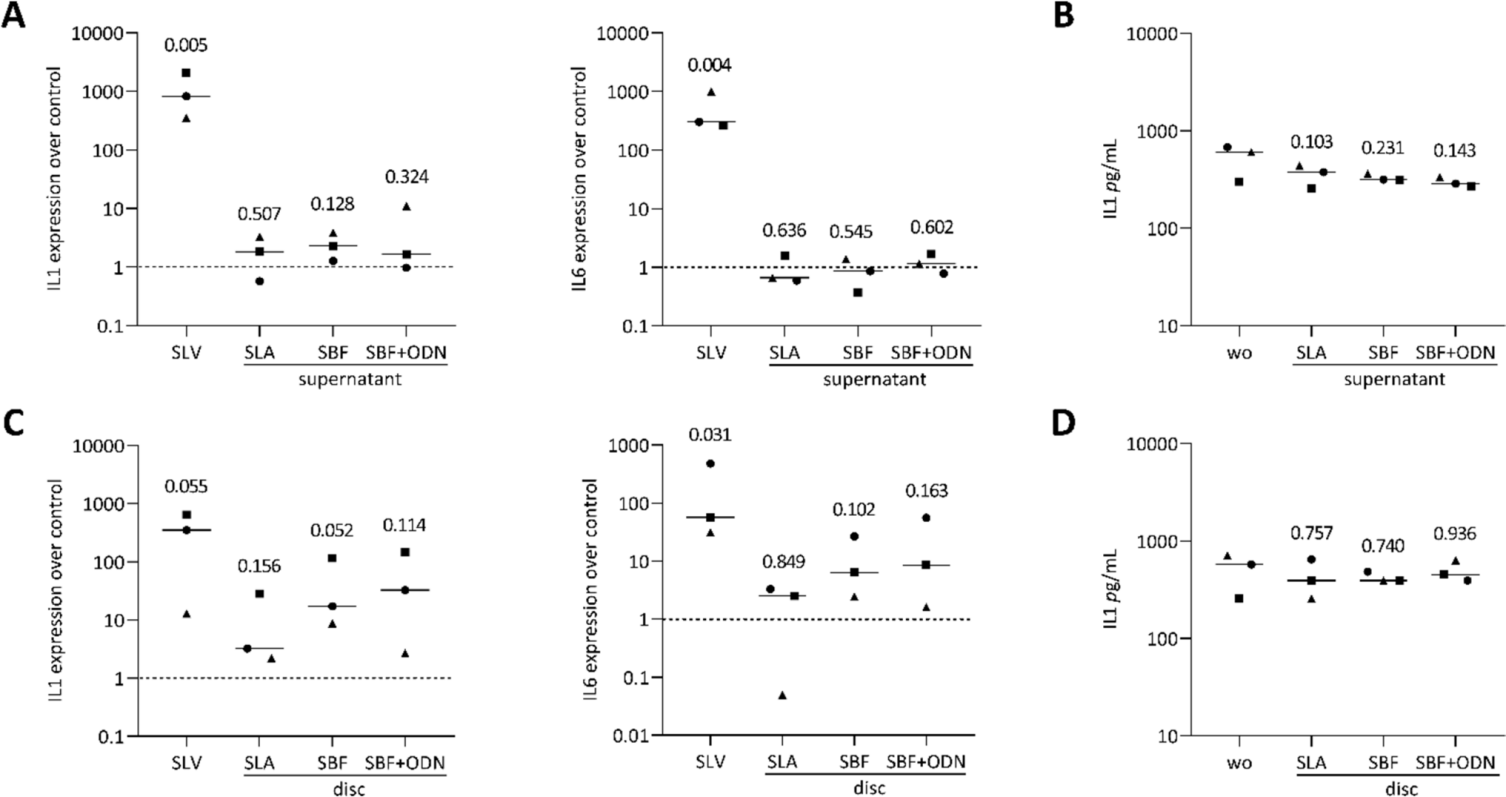
Macrophages were exposed to the supernatant (**A**) or grown in the presence of titanium discs (**C**) of each group and 5% saliva (SLV) as positive control. Results were normalized for untreated cells for expression changes. (**B**, **D**) Immunoassay indicated basal IL1 levels in pg/mL of untreated cells. Different symbol shapes represent independent experiments. Statistical analysis was performed using ratio-paired t-tests compared to untreated controls, and p-values are shown

Next, we investigated the potential of supernatants and titanium discs to induce an inflammatory response in macrophages which can be stimulated by saliva [44, 45]. Upon culture with titanium discs, we observed a modest increase in IL1 and IL6 gene expression (Fig. 9C), accompanied by a slight increase in IL1 expression in cells exposed to the supernatants (Fig. 9A). However, these changes did not correspond to the IL1 protein level changes at the two experimental conditions (Fig. 9B - D). Overall, the results suggest that, again, it is the direct contact of the cells with the discs that trigger a moderate inflammatory response with no significant impact on the surface coatings.

### Odanacatib coating inhibits cathepsin k without impacting osteoclastogenesis

**Fig. 10:**
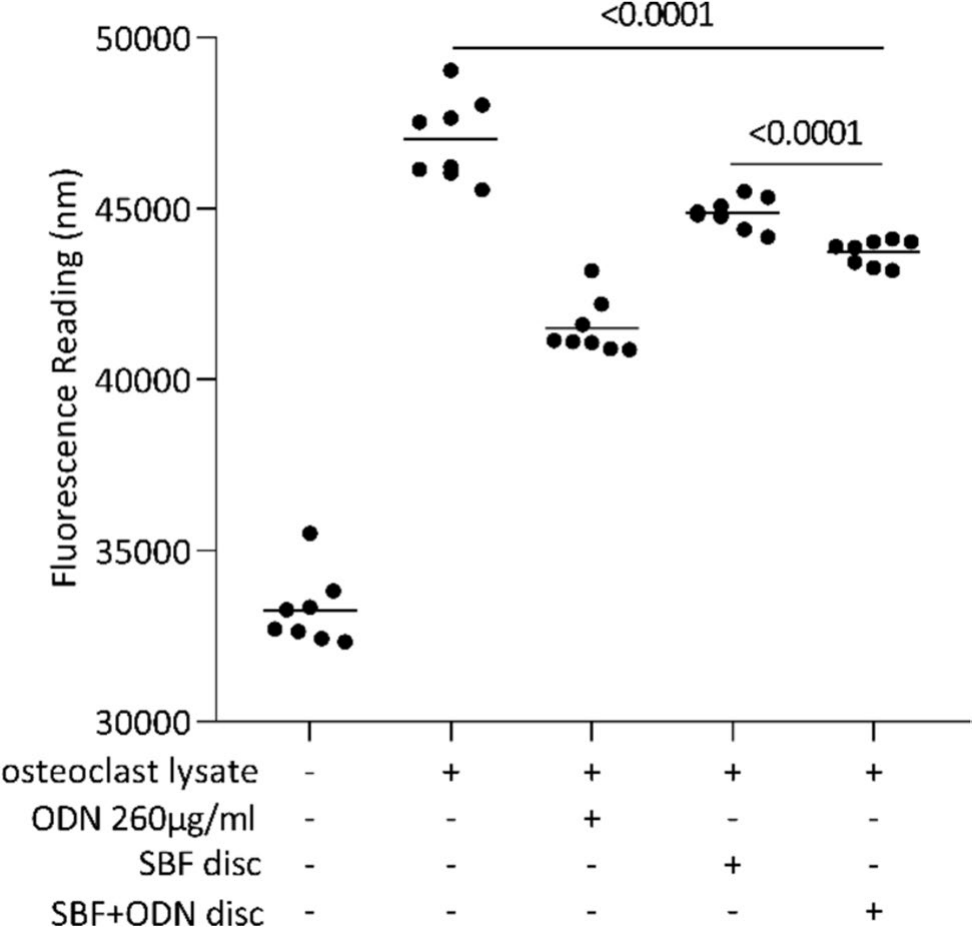
Cathepsin K activity was conducted with titanium discs coated with SBF and SBF + ODN, in the presence of osteoclast lysates. Fluorescence intensity indicating CATK activity was determined. Statistical analysis was performed using ratio-paired t-tests. Notably, the presence of ODN significantly lowers the CATK activity intrinsic to the osteoclast lysate

**Fig. 11:**
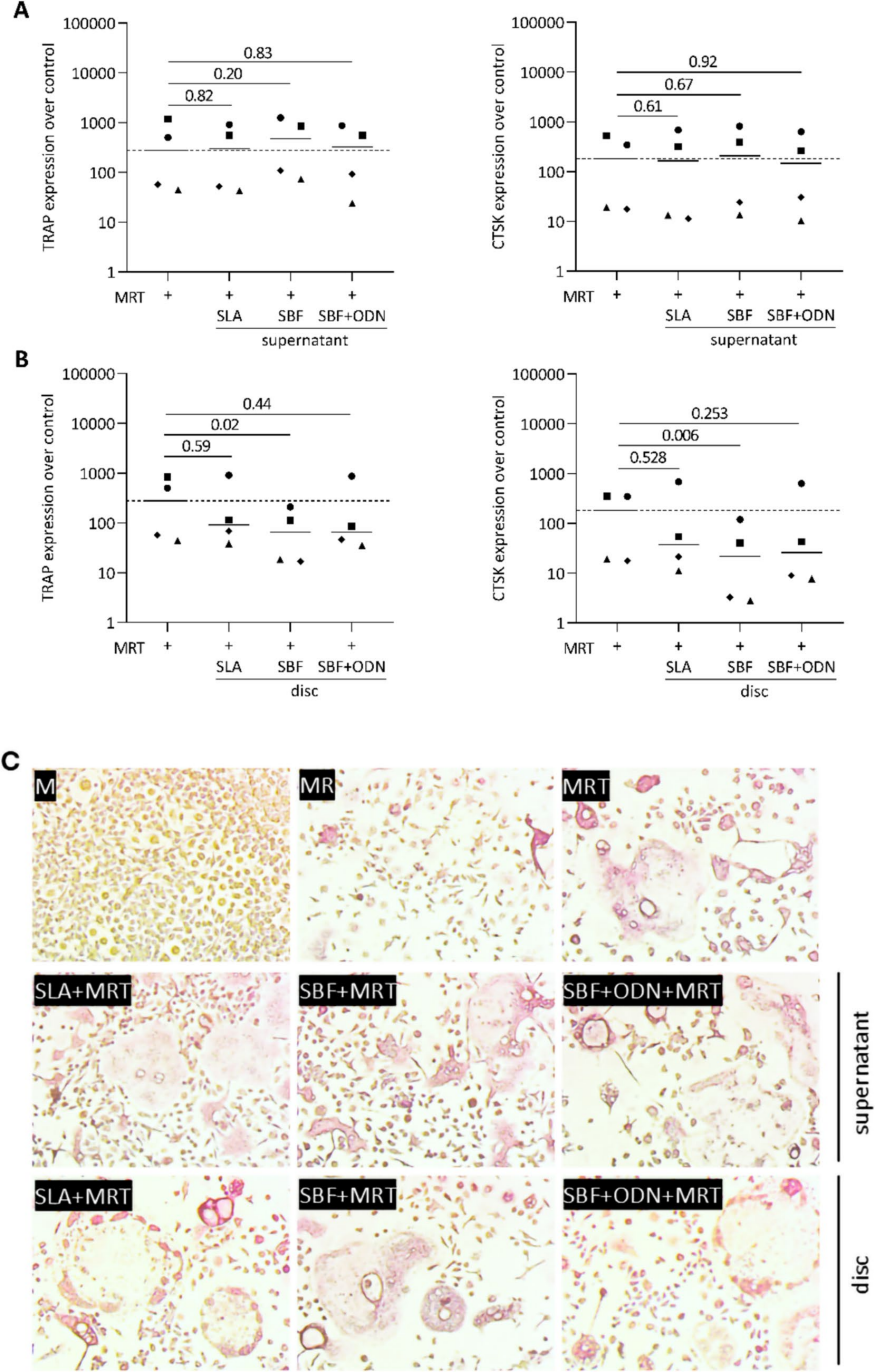
Bone marrow-derived cells were exposed to supernatants (**A**, **C**) and titanium discs (**B**, **C**) from each group in the presence of M-CSF, RANKL, and TGF-β (MRT). **A**, **B** Gene expression levels of TRAP and CTSK were normalized to untreated cells. Different symbol shapes in the graphs represents independent experiments. The SBF coating resulted in decreased TRAP and CTSK expression, but this was not reflected in TRAP staining. Statistical analysis was performed using ratio-paired t-tests, with p-values indicated. **C** Representative TRAP-stained images show that osteoclast formation was not significantly suppressed by SLA, SBF, or SBF + ODN disc and respective supernatants

We next sought to clarify the activity of ODN coating on a titanium disc. Here, we establish a bioassay to measure the CATK activity using a fluorogenic substrate. Our results demonstrated that the CATK activity of the osteoclast lysate was reduced in the presence of SBF + ODN discs, suggesting that ODN remains active upon coating (Fig. 10). As expected, the ODN coating did not affect osteoclastogenesis or osteoclast survival, as indicated by unchanged levels of TRAP and CATK gene expression (Fig. 11A, B), which was further confirmed by TRAP staining (Fig. 11C). However, SBF coating decreased osteoclastogenesis.

## Discussion

Despite its widespread use in oral rehabilitation, research on dental implants still faces unresolved issues, particularly concerning implant failure despite good oral hygiene [46, 47]. This observation becomes even more important when considering that the implant sources are not exclusively covered with osteogenic cells, particularly with immediate implant placement; today's progressive protocols, soft tissue cells from the gingiva are likely to come into contact with the implant surface – and experience its impact. One hypothesis suggests that releasing titanium ions and particles during implant insertion and healing may trigger local cellular responses, including reactions from foreign bodies [48]. In the normal peri-implant environment, not only osteogenic cells but also local fibroblasts, epithelial cells, and macrophages can potentially exhibit a mild inflammatory phenotype, releasing cytokines that may contribute to chronic inflammation [5]. Within this context, gingival fibroblasts offer critical insights into connective tissue responses to inflammation and healing [25, 49], while HSC2 cells guide the understanding of epithelial cell behavior and pathology [27]. The inflammatory response initiated by the early interactions between cells and the titanium surface, although not entirely understood, is critical for osseointegration and the overall soft tissue healing response. Our research focused on how titanium discs with various modifications affect viability and, in this context, cytokine expression in gingival fibroblasts, HSC2 cells, and murine macrophages using an in vitro bioassay approach. Our results partially support the hypothesis that implant surfaces have an impact on cell viability and increase basal cytokine expression, as indicated by elevated levels of IL8 and IL1. These alterations were influenced more by growing cells in the presence of the titanium discs rather than by the respective supernatants, while surfaces coating with SBF or ODN had no significant influence on cytokines expression. Thus, the changes observed in changes observed are presumably associated with the alterations in cell viability when cells are grown on the surface of the titanium discs.

To contextualize our findings within the broader understanding of cellular response to titanium discs, our observations are consistent with the evolving paradigm highlighting the potential impact of a titanium surface modification on cell viability. Consistently, a significantly lower formazan formation was noticed when gingival fibroblasts and MG63 osteogenic cells were cultured on SLA discs compared to a regular tissue culture plate [50, 51]. A similar reduction in cell viability was observed with osteogenic cells cultured on titanium discs with surface modifications, including hydroxyapatite/tricalcium phosphate coatings [47, 48], but also in murine mesenchymal cells exposed to SBF coating [50]. Additionally, supernatants from long-term exposure to titanium discs lowered osteogenic cell viability [49] Together, these findings support the notion that growing cells on titanium discs may trigger cell death, at least in vitro. Importantly, there is a causal link between cell death and the initiation of an inflammatory response; thus, dying cells can trigger an inflammatory response [9, 42], apart from causing other local reactions in gingival fibroblasts [52]. It was, therefore, logical to investigate the impact of the titanium discs on the expression of indicatory inflammatory cytokines IL8 and IL1.

Building on the assumption that cell death is linked to an inflammatory response, it was obligatory to determine the effects of titanium discs on cytokines expression. Our observations are consistent with others; for instance, SLA discs moderately increased IL8 but decreased IL6 expression compared to standard tissue culture surfaces in gingival fibroblasts; however, these changes did not translate into protein levels [8]. In our culture setting, we observed an increase in IL8 expression that, however, caused protein level changes in gingival fibroblasts and HSC2 cells. Experiments conducted with titanium disc supernatants further supported increased IL8 expression in both cell types [28]. Our supplementary data with gingival fibroblasts and HSC2 cells indicate that titanium discs moderately elevate other inflammatory markers, such as CXCL1, CXCL2, CXCL10, and IL6; however, these changes were observed in only two out of three independent experiments. Nonetheless, integrating our results with existing knowledge [7] suggests a low inflammatory response in gingival fibroblasts when cultured with titanium discs and, to some extent, with the supernatants. Our observations with HSC2 cells resembling an oral epithelial cell phenotype add a new aspect to the overall concept of an adverse reaction to titanium surfaces that are currently based on macrophages.

Among the first cells to interact with implant surfaces, macrophages play a crucial role in regulating the immune response to biomaterials and are supposed to be the primary target cell for titanium particles and ions released from implants [19]. In the present study using primary murine macrophages, we observed a minor increase in IL1 and IL6 expressions on disc cultures, with a slight increase in IL1 expression caused by the supernatants; however, these changes did not translate into protein-level alterations. Nevertheless, our finding points in the same direction as previous research reporting increased IL1 expression caused by SLA surfaces in RAW 264.7 murine macrophages [20]. However, and even though ODN can inhibit inflammation and bone loss in endodontic disease by reducing IL6 and TNFα in macrophages [33, 53], in our experiments, IL1 and IL6 expression by macrophages remained unchanged by SBF + ODN coating or the equivalent supernatant. Thus, it is mainly the material or the SLA treatment rather than the SBF and ODN surface coating that triggers the moderate cytokine expression by the murine macrophages.

The SBF coating has a minimal impact on osteoclasts, resulting in lowering osteoclastogenesis. Previous studies have shown that while titanium surfaces coated with SBF exhibit reduced osteoclastic activity, they do not entirely prevent osteoclast formation, and the overall response remains unclear [54]. It is now interesting to evaluate how effectively ODN, when released from titanium discs, inhibits CATK activity. Our results confirm that ODN remains effective in inhibiting CATK when released from titanium discs, as demonstrated by its inhibition of the activity of CATK in osteoclast lysates. However, ODN targets selective inhibition of the CATK rather than osteoclastogenesis [17]. As a result, the ODN-coated surfaces did not impact osteoclastogenesis, with levels of gene expression of TRAP and CATK remaining unchanged. This suggests that although the ODN acts on inhibition of the activity of the CATK, it does not interfere with the formation of new osteoclasts [16]. Taken together, our findings are consistent with data indicating that while ODN reduces osteoclastic bone resorption, ODN does not alter the expression of TRAP and CATK markers associated with osteoclast formation [54]. This suggests that ODN's role is specific to reducing bone resorption rather than affecting the formation of new osteoclasts.

The study has limitations that could guide future research. Firstly, the effects of titanium discs, particularly growing cells on titanium discs, on cytokines expression by gingival fibroblasts and HSC2 cells are relatively low, not to be compared with the response of cells to the IL1 and TNFα exposure [35], but significant. Also, measuring a few cytokines is representative for the overall cell response; thus, future research should include RNAseq approaches to understand signature genes of the transcriptome. Moreover, the underlying mechanisms of the inflammatory response remain unclear – but there is a plausible link to the dying of the cells, which can be a trigger for cytokine expression [9, 42, 52].Thus, since gingival fibroblasts and HSC2 cells are not classical phagocytic cells, the increased IL8 expression might be related to cell damage and toxicity. Further evidence supporting a potential inflammatory response due to toxicity comes from observations of dose-dependent toxicity of titanium and zirconia particles on periodontal fibroblasts [29]. However, it remains uncertain whether the in vitro findings with gingival fibroblasts and HSC2 cells can be translated into clinical scenarios because we have exposure of discs to overnight period, which simulated a possible early release of particles and ions. A more refined understanding is needed to map which and how many of the compounds are released into the medium, how this release changes over time, and which other conditions, such as pH and temperature, may affect this process. Nevertheless, when we check the “pellet” of the supernatant, we saw particles in the microscope, but these observations were more impressions rather than scientific findings. Analytical techniques to measure the release of particles and ions should be considered in future studies.

Further limitations are that, apart from the missing analytics, it is unclear what causes the soft tissue cells death and the inflammatory response we have identified on the discs cultured. Therefore, future research should refine the model and consider toxicity as a potential cause for achieving a moderately inflammatory phenotype, particularly evaluating the context of necroptosis. The same consideration applies to osteoclastogenesis, which was not affected by the titanium discs and their coatings; nonetheless, titanium nanotopography does impact osteoclastogenesis in vitro [55]. Perhaps more relevant is to reemphasize that, in vitro, the classical titanium discs with a standard surface modification can trigger cell death – at least when compared to a standard tissue culture surface. Somehow, surprisingly, this observation remains a phenomenon, and the underlying reasons are unclear. Imaginably, this simple life-dead staining and the expression of cytokines becomes a screening approach to identify cell-friendly implant surfaces. Another important aspect to consider is the exposure time, which falls within the 12–48-h range. This duration represents the early phase of inflammation, sufficient for detecting inflammatory events triggered by the direct contact of cells grown on the titanium surface or in the presence of their respective supernatants. Therefore, conclusions should be limited to short-term exposure, and it cannot be excluded that, over time, the cells may adapt to the surface and exhibit reduced cytokine expression.

Additional limitations are that we did not include titanium discs with a polished surface compared with the SLA modifications, particularly because the rough surface, independent of the SBF coatings, might have provoked a moderate inflammatory response. This would have allowed us to distinguish the material properties from the surface modifications. The regular titanium discs were not considered as controls as the original idea was to study the impact of SBF and it contains ODN. This coating did not significantly affect the cell response. Nevertheless, there is a small trend toward induction of cytokine expression by the supernatants of the discs; thus, the tissue culture plate is a proper control in this setting. Again, we cannot rule out that also supernatants of regularly polished titanium discs show a similar trend; theoretically, if this is the case, it is the material properties rather than the surface modification triggering the cell response. Building on this, although the CatK kinetic bioassay confirmed the activity of ODN on the titanium surface, the release and precise concentration of ODN on the surface were not quantified. These limitations stem from the biomimetic protocol, which involved a four-step deposition process with daily renewals of the SBF + ODN solution to maintain consistent concentration. While this ensured uniform deposition, quantifying the release and concentration kinetics over longer periods would be essential for validating the findings. Such analyses would not only enhance the understanding of this biomimetic protocol for coating bioactive compounds but also provide valuable insights into the behavior and stability of ODN on titanium surfaces over time.

## Conclusion

In summary, our in vitro experiments use simulation patterns to replicate complex organisms exposed to either to supernatants or the surfaces of titanium, which do not fully reflect clinical scenarios. Consequently, while our results represent short time observations, a significant question remains between these experimental models and clinical outcomes. Our study reveals that certain titanium disc modifications, such as SLA, moderately influence cytokine expression and inflammatory markers in vitro, but the clinical relevance of these findings is uncertain due to the limitations of the experimental models. Future research should focus on refining these models, exploring various coating and alloy methods, and evaluating their effects during both the early and late stages of implant interactions. Addressing these aspects is crucial for bridging the gap between experimental results and clinical applications, ultimately optimizing implant design and enhancing long-term clinical success.

## Supplementary Information

Below is the link to the electronic supplementary material.

ESM 1 (7.10 MB)

## Data Availability

No datasets were generated or analysed during the current study.
