# Supplementary material for: Cytokine expression of soft tissue cells cultured with titanium discs and their respective supernatants in vitro
Source: Clin Oral Investig. 2025 Jan 14;29(1):62. doi: 10.1007/s00784-024-06123-1 (PMC11732886; doi:10.1007/s00784-024-06123-1)
Supplement: Supplementary file 1 — (7.10 MB) [file 784_2024_6123_MOESM1_ESM.docx]

**Supplementary**

**B**

**A**

***
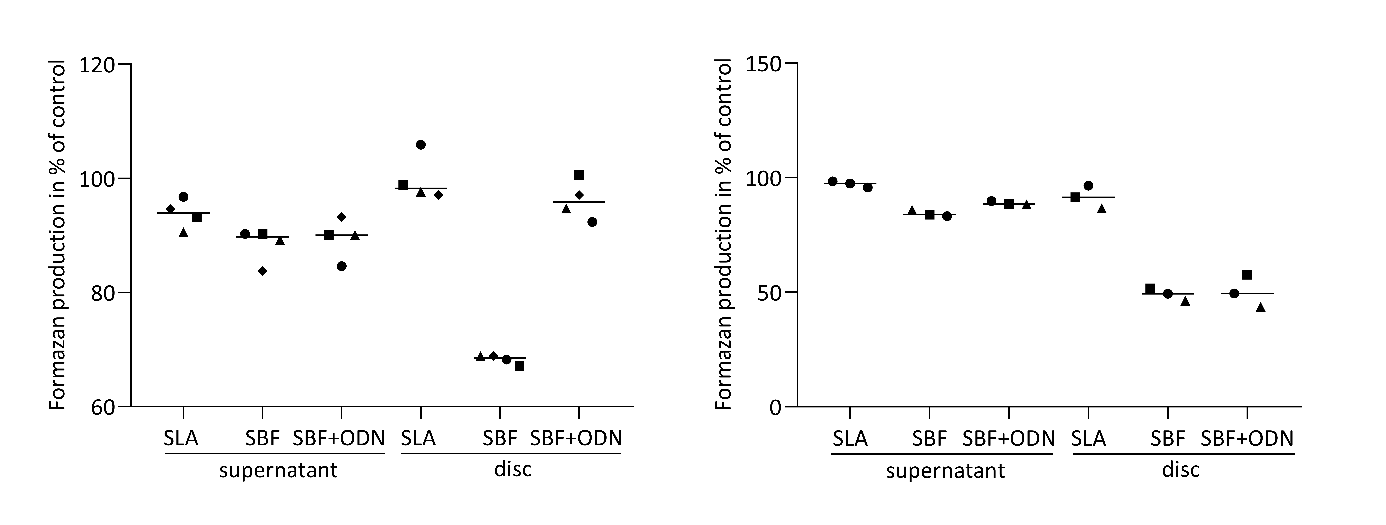
***

***Fig. 1*.** Cell viability assays were performed on human gingival fibroblasts (A) and HSC2 cells (B) stimulated with the supernatants and discs from each group—SLA, SBF, and SBF+ODN overnight. Cell viability was determined by measuring formazan production, with results presented as a percentage compared to the unstimulated controls, which are considered as 100% viability. The findings revealed a notable decline in viability for gingival fibroblasts and HSC2 cells in direct contact with the titanium discs. Conversely, exposure to the supernatants resulted led to only a slight decrease.

*Odanacatib enhanced wound closure in* *oral squamous cell carcinoma*

We performed a classic scratch test to simulate a wound-like scenario that requires proliferation and migration in gingival fibroblasts and oral squamous cell carcinoma. Overall, the wound closure response was significantly more pronounced in squamous cell carcinoma cells than in gingival fibroblasts. Specifically, in gingival fibroblasts, wound closure remained unaffected by exposure to the supernatants and titanium discs with SLA, SBF, and SBF+ODN coating. However, squamous cell carcinoma showed significantly enhanced wound closure when exposed to the supernatants and SBF+ODN-coated discs compared to the other treatments and the serum-free medium (Figure 1). Furthermore, the SBF alone had a less pronounced impact on wound closure. Our findings suggest that the addition of ODN to the titanium surface coating supports wound closure in oral squamous cell lines.

*
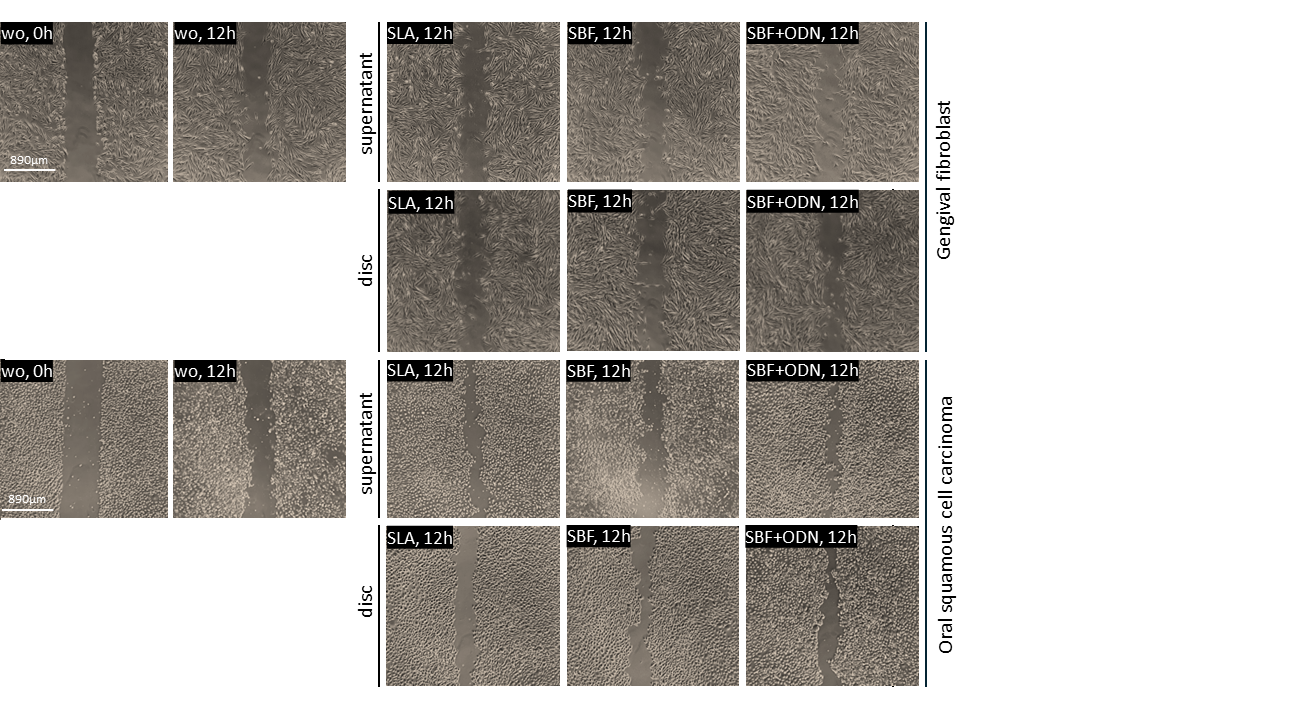
*

***Fig. 2*.** Gingival fibroblasts and oral squamous cell carcinoma were grown to confluence and then subjected to a scratch assay. After removing debris, the cells were treated with supernatants and titanium discs from each group—SLA, SBF, and SBF+ODN. Images were captured immediately after the scratch, 0 hours, and after 12 hours of incubation. The results support that the supernatant and the SBF+ODN-coated disc enhanced wound closure, showing a smaller gap in oral squamous cell carcinoma cells. The scale bar represents 890 μm.

*
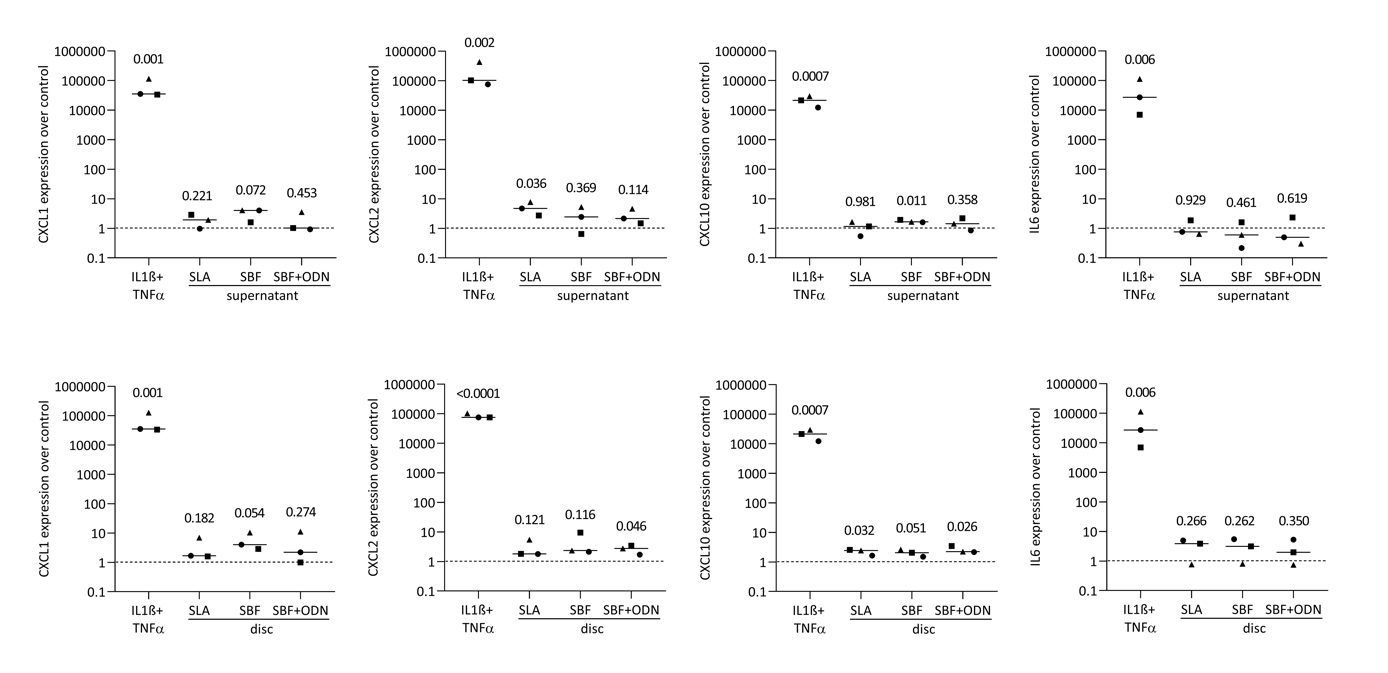
****Fig. 3*.** Gingival fibroblasts were exposed to the supernatants or grown in the presence of titanium discs of each group and IL1β+TNFα as positive controls. Results were normalized for expression changes for untreated cells. Different symbol shapes represent independent experiments. Statistical analysis was performed using ratio-paired t-tests compared to untreated controls, and p-values are shown.

*
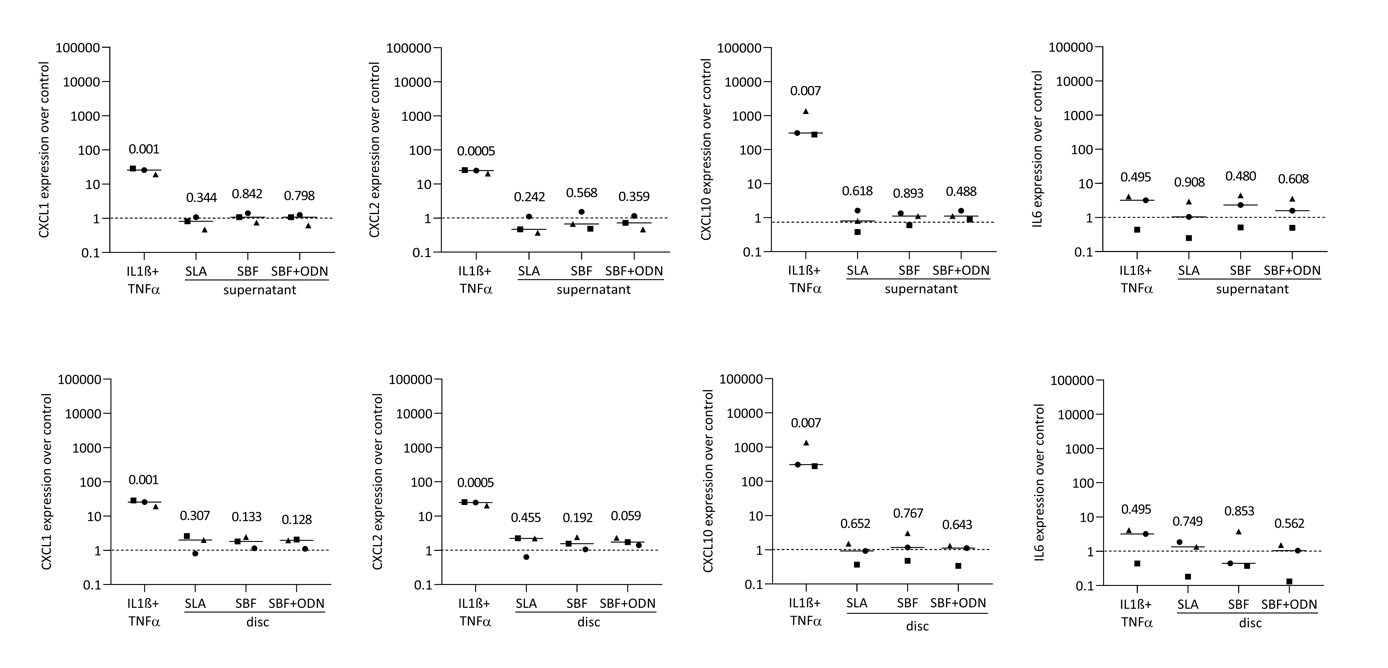
****Fig. 4****.* HSC2 cells were exposed to the supernatants or grown in the presence of titanium discs of each group and IL1β+TNFα as positive controls. Results were normalized for expression changes for untreated cells. Different symbol shapes represent independent experiments. Statistical analysis was performed using ratio-paired t-tests compared to untreated controls, and p-values are shown.


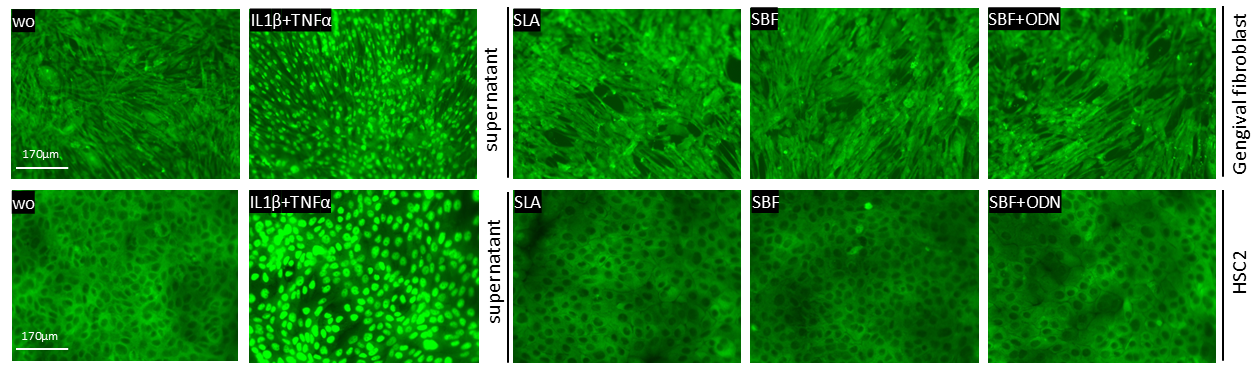


***Fig. 5.*** Immunofluorescence staining for nuclear translocation of p65 in gingival fibroblast and HSC2 cells. Cells were exposed to supernatants of titanium discs of each disc from each group—SLA, SBF, SBF+ODN, and IL1β+TNFα as positive control. Without (wo) is the serum-free medium alone. The supernatants did not induce a positive signal of nucleus translocation in gingival fibroblast and HSC2 cells. The scale represents 170 μm.


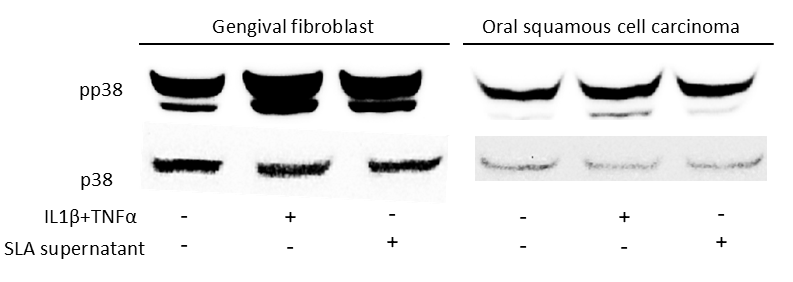


***Fig. 6.*** SLA supernatant increased phosphorylation of p38 in gingival fibroblasts. Western blot analysis was performed on gingival fibroblasts and HSC2 cells for phospho-p38 and total p38. Cells were treated with supernatant from the SLA and IL1β+TNFα as positive controls. "wo" means without and represents unstimulated cells.
